# Integration of multimodal data in the developing tooth reveals candidate regulatory loci driving human odontogenic phenotypes

**DOI:** 10.3389/fdmed.2022.1009264

**Published:** 2022-11-30

**Authors:** Emma Wentworth Winchester, Alexis Hardy, Justin Cotney

**Affiliations:** 1 DMD/PhD Program, University of Connecticut School of Dental Medicine, Farmington, CT, United States,; 2 Master of Genetics Program, Paris Diderot University, Paris, France,; 3 Department of Genetics and Genome Sciences, University of Connecticut School of Medicine, Farmington, CT, United States,; 4 Institute for Systems Genomics, University of Connecticut, Storrs, CT, United States

**Keywords:** odontogenesis, gene regulation and expression, enhancer activation, chromatin regulation, single cell RNA analysis, multiomic analysis

## Abstract

Human odontogenic aberrations such as abnormal tooth number and delayed tooth eruption can occur as a symptom of rare syndromes or, more commonly, as nonsyndromic phenotypes. These phenotypes can require extensive and expensive dental treatment, posing a significant burden. While many dental phenotypes are heritable, most nonsyndromic cases have not been linked to causal genes. We demonstrate the novel finding that common sequence variants associated with human odontogenic phenotypes are enriched in developmental craniofacial enhancers conserved between human and mouse. However, the bulk nature of these samples obscures if this finding is due to the tooth itself or the surrounding tissues. We therefore sought to identify enhancers specifically active in the tooth anlagen and quantify their contribution to the observed genetic enrichments. We systematically identified 22,001 conserved enhancers active in E13.5 mouse incisors using ChIP-seq and machine learning pipelines and demonstrated biologically relevant enrichments in putative target genes, transcription factor binding motifs, and in vivo activity. Multi-tissue comparisons of human and mouse enhancers revealed that these putative tooth enhancers had the strongest enrichment of odontogenic phenotype-associated variants, suggesting a role for dysregulation of tooth developmental enhancers in human dental phenotypes. The large number of these regions genome-wide necessitated prioritization of enhancer loci for future investigations. As enhancers modulate gene expression, we prioritized regions based on enhancers’ putative target genes. We predicted these target genes and prioritized loci by integrating chromatin state, bulk gene expression and coexpression, GWAS variants, and cell type resolved gene expression to generate a prioritized list of putative odontogenic phenotype-driving loci active in the developing tooth. These genomic regions are of particular interest for downstream experiments determining the role of specific dental enhancer:gene pairs in odontogenesis.

## Background

Dental developmental malformations range from rare syndromes such as amelogenesis or dentinogenesis imperfecta (1–5) to relatively common nonsyndromic phenotypes such as abnormal tooth number (6–10) or enamel hypoplasia (11, 12). Aberrations from normal dental development may predispose individuals to dental disease (13, 14) and may require costly and complex interventions. As dental health is known to impact systemic health (15, 16), abnormal dental development poses a substantial public health burden and determining causes of specific phenotypes is a priority. While environmental factors may play a role in the incidence of many of these nonsyndromic phenotypes, the heritability of normal and abnormal dental phenotypes has been reported to range from 40% to 90% (17–23). This suggests a strong genetic component, however the portions of the human genome contributing to these findings have not been comprehensively identified.

In most mammals the primary teeth begin to develop during early embryogenesis and hard dental tissues are formed prior to tooth eruption into the oral cavity shortly after birth. Both the patterning of tooth sites during early dental development, which determines tooth number, and the formation of tooth roots during late dental development, which results in tooth eruption, occur primarily *in utero*. As such, the underlying genetic causes of many dental malformations and diseases likely exert their effects during development. Therefore, these genetic programs that coordinate this process are of particular interest in identifying drivers of dental malformations. While rare syndromes affecting teeth have been linked to mutations in specific genes (24–27), most cases of common dental phenotypes such as abnormal tooth eruption (28) and tooth number (29–31) have not been linked to large DNA copy number changes or deleterious mutations in single genes. Their relatively high heritability, lack of common causal mutations in protein-coding genes, and the tooth-isolated nature suggests that risk of these phenotypes may instead be due to defective gene regulation caused by variation in gene regulatory regions.

Enhancers are sequences enriched with transcription factor binding motifs that can regulate target gene expression in spatiotemporal-specific contexts over long genomic distances (32–36). Disruption of enhancers can modulate gene expression when and where that enhancer is normally active, typically resulting in tissue-isolated abnormalities (35, 37, 38). Common human single nucleotide polymorphisms (SNPs, or variants) associated with craniofacial phenotypes such as nonsyndromic orofacial clefting and craniofacial morphological variation are enriched in enhancer sequences active in embryonic human craniofacial tissues. These genome-wide interrogations of thousands of enhancers across hundreds of tissues have thus illustrated that craniofacial enhancers specifically contribute to craniofacial phenotypes (39–41). However, findings in the bulk human face have not been extended to investigate the contribution of craniofacial or dental enhancers in dental morphogenesis or disease. As a consequence of this, enhancers have remained largely unstudied in the developing tooth.

Ideally, identification and characterization of such enhancer sequences would use primary developing human teeth. However, human dental development is a long process; development of the primary teeth begins at 6 weeks gestation and is not complete until several years after birth. Due to the gestational time period of dental patterning and morphogenesis (42–46) and lack of clear human *in vitro* models, tooth development is primarily studied using mice. The early stages of development of the mouse dentition are largely morphologically and molecularly analogous to human dental development (42–44), and many rare human dental syndromes are modelable in mice (42, 44, 47–49). While a group has previously identified a region upstream of *Shh* whose disruption demonstrates a dental phenotype in mice, systematic characterization of the activity of the noncoding genome in the developing tooth of mammals has not been performed (35, 50). As such, the role of enhancers in odontogenic phenotypes and the conservation of the noncoding drivers of dental development between human and mouse genome-wide has not been investigated.

Here we demonstrate that craniofacial enhancers active in bulk human craniofacial tissue and conserved in mouse are enriched for variants associated with dental developmental phenotypes. We extended these findings by identifying and validating enhancers of the developing cap stage tooth with a well-established ChIP-seq and chromatin state annotation pipeline (40, 51, 52). We showed that enhancers active in the cap stage mouse incisor have the highest burden of odontogenic phenotype-associated variants compared to all other tissues, indicating a role for dental enhancers in human odontogenic phenotypes. We further prioritized these enhancer loci for future investigations based on the predicted importance of their putative target genes in dental development. To do this we leveraged multiple data modalities including bulk RNAseq, co-expression network analysis, single cell RNAseq, previously generated Genome-Wide Association Study (GWAS) data, and ChIPseq-based enhancer calls. We present the results of this extensive analysis as a prioritized list of gene loci whose enhancers are predicted to contribute to common, nonsyndromic human odontogenic aberrations. We expect this list to be useful for future in-depth experiments profiling specific regions and their effect on normal dental development in mice. Additionally, the tooth-specific enhancers we identified here are of particular interest as they provide precise molecular tools for tooth specific knockouts and reporter gene expression, as we have previously described for specific regions of the mouse brain (53).

## Materials and methods

### Chromatin immunoprecipitation and chromatin state segmentation

Cap stage E13.5 mouse mandibular incisors were isolated bilaterally from ~10 mixed sex C57BL/6J *Mus musculus* embryos (Jackson Laboratories), pooled, and crosslinked using 1% formaldehyde as previously described (54, 55). Animals were raised and sacrificed in compliance with IRB approval (protocol AP-200061-0723). Upon isolation of nuclei and shearing, soluble chromatin was divided equally across multiple tubes for immunoprecipitation as previously described (104, 105) with antibodies against H3K4me1 [Abcam ab8895 (Lot GR276935-1)], H3K4me2 [Abcam ab7777 (Lot GR102810-2)], H3K4me3 [Abcam ab8580 (Lot GR273043-2)], H3K27ac [Abcam ab4729 (Lot GR276930–1)], H3K27me3 [Millipore 07–449 (Lot 2736613)], and H3K36me3 [Abcam ab9050 (Lot GR273247-1)]. For tissue availability purposes, a single replicate of each mark was obtained. Subsequent sequencing, alignments, imputation of signals, and chromatin state segmentation using 15 and 18 state models were performed as previously described (40). Raw Fastq files and *p*-value tracks for these chromatin marks can be found on GEO at GSE197645. The same chromatin signals were retrieved from all available data in mouse ENCODE (56) for multiple replicates of 12 tissues and 10 timepoints and imputed to yield 1316 distinct chromatin signals. These imputed signals were then segmented with the same models as above resulting in 154 chromatin state segmentations (https://genome.ucsc.edu/s/emmawentworth/mm10_all_18state). Tooth specific enhancers were identified using bedtools [v2.29.0, (57)] on strong enhancer annotated segments [18 State chromatin segmentations; States 8, 9, 10; (52)] and compared to other non-craniofacial mouse tissues (samples without any dental tissue) and the same chromatin state segments. Mouse strong enhancers were converted to hg19 coordinates (kent-tools, v1.04.00; minimum sequence conservation 25%) for GWAS analyses (see below). Target genes of these enhancers were identified using rGREAT [v1.26.0, (58, 59)] for mm10, using the default settings.

### GWAS enrichment analysis

Crohn’s disease, tooth eruption, and odontogenesis-associated variants were obtained from GWAS catalog (EFO_0000384, GO_0044691, GO_0042476). Variants identified in eastern european cohorts with *p* ≤ 5 × 10^−8^ were retained for each category. GREGOR [v1.4.0, (60)] was used to interrogate enrichment of disease-associated variants within strong enhancers (18 state segmentation, states 8–10) of each biosample, with the control group of Eastern European background variants. Human enrichment analysis was performed on 18 state segmentations of published samples obtained from (40, 52) and 127 human cell types profiled by Roadmap Epigenome (51, 61). Mouse enrichment analyses were performed on 18 state segmentations from published mouse tissues from the ENCODE project and our E13.5 mouse incisor sample, as described above.

### Bulk RNA-seq

Bulk RNA-seq fastqs from bulk craniofacial samples (*n* = 4; E13.5, E14.5) from (56), molars (*n* = 30; 3-E12.5, 7-E13, 7-E14, 3-E15.5, 7-E16, 3-E17.5) and incisors (*n* = 3, E12.5) from (62, 63) were obtained from GEO and aligned using Kallisto (kallisto v0.46.2) to the mm10 genome (64). Aligned libraries were imported to DESeq2 (v1.26.0) (65) using tximport (v1.14.2) (66) and one replicate of E13 was omitted as an outlier. Genes were filtered for minimum transcript count of 5 in at least 3 replicates. To obtain differentially expressed genes of the cap stage tooth, we used DESeq2 with suggested parameters on non-batch corrected E14 molars (7 replicates) compared to E13.5 and E14.5 bulk craniofacial processes (4 replicates). Genes with a log fold change greater than 2 in the tooth and with a Bonferroni adjusted *p* value of ≤0.05 were retained and considered differentially expressed. For WGCNA, only tooth samples were used. Batch effects were corrected by removing PC1 and PC2 as sources of variation using RUVseq (v1.20.0) (67). The remaining 32 samples were processed using DESeq2 for WGCNA input. Variance stabilized transformation count matrix for all samples were imported into a WGCNA pipeline (v.1.70-3) (68). In brief, samples were filtered for outliers and genes were filtered for quality. Power for each file was obtained and a threshold of 0.85 was used to define power of 8. WGNCA network was built using unsigned TOM, minimum module size of 50, gene dendrogram merge cut height of 0.25, and a deepSplit of 2. Correlation and intramodule connectivity for each module was identified. Gene ontology enrichment analysis and subsequent figures for DEGs and WGNCA modules were generated using EnhancedVolcano, GOplot, clusterProfiler, with “enrichGO” and “enrichDGN” modalities on genes and their 1 : 1 human orthologs (clusterProfiler v3.14.3, org.Hs.eg.db v3.14.0, org.Mm.eg.db v3.10.0, EnhancedVolcano v1.4.0, GOplot v1.0.2) (69–73).

### Single cell RNA-seq

Raw fastqs were retrieved from GEO (GSE142201) (62) and aligned to the mm10 genome (GRCm39) using Kallisto/bustools (kallisto v0.46.2, bustools v0.40.0) (64), and Kallisto for bulk RNA-seq libraries. Kallisto/bustools was used in filtering and Seurat object generation. Seurat (v3.2.0) (74) was used to merge replicates. Cells were filtered (<5% mtDNA, >200 nFeatureRNA) and log normalized. Cell cycle stages were assigned with mouse orthologs from known human cell cycle genes. Data was scaled using all genes. Nearest neighbors were assigned using dimensions 1 : 15 *via* Louvain algorithm. Clusters were generated at resolution = 0.3. UMAPs were generated with dimensions 1 : 15. Epithelial analysis was clustered at resolution = 0.2, using dimensions 1 : 15. Marker genes were identified with DESeq2 using logfc > 0.5, min.pct = 0.25. SymIDs were converted to EntrezID with clusterProfiler. clusterProfiler was used to calculate GO term enrichment against background. clusterProfiler “simplify” combined similar GO terms, with z score cutoff of 0.5. Marker genes of annotated cell types were identified after dropping all cells with the identity “Bone Progenitor Cells” from analyses, using DESeq2 with logfc > 1.0, min.pct = 0.25, max.cells.per.ident = 400.

### Marker gene validation

In situ hybridization images for EK marker gene validation were obtained from the GenePaint online resource and a literature search for specific genes in the developing tooth (74–79).

### Region prioritization

To prioritize loci of potential odontogenesis, we looked at the relevance of putative target genes for tooth enhancers. All genes from WGCNA analyses, excepting genes belonging to the grey module (low expression module), were considered for prioritization. Genes were given a “score” based upon a number of factors. Genes were given one point for being a marker gene for any scRNA-seq annotated cell type. An additional point was given for marker genes of dental-specific cell types (Enamel Knot, Epithelium, Mesenchyme, and Perivascular Cells). We obtained hg19 coordinates of GWAS variants associated with craniofacial and dental phenotypes (EFO_0009892, EFO_0600095, GO_0044691, EFO_0009331, Orphanet_141136, and HP_0000175) and identified human genes located within 500 kbp of at least one variant (Supplementary Tables S22, S23). Mouse orthologs of these genes were given an additional point. Genes were given additional points based on the number of TSEs predicted to be associated by GREAT: 0 TSEs = 0 points, 1–2 TSEs = 1 point, 3–4 TSEs = 2 points, 5–6 TSEs = 3 points, 7+ TSEs = 4 points. Genes belonging to tooth-relevant WGCNA modules (Figure 2E) were given additional points for being enriched for TE target genes, dental phenotypes ontology categories, and DEGs. We used mm10 coordinates of all VISTA + tooth enhancers to identify all genes within 1Mb of each enhancer (Supplementary Table S24). These genes near VISTA validated enhancers were given a point. Genes which were predicted to be targeted by at least one tooth enhancer were given an additional point. Lastly, genes which also appeared as a cap stage tooth-specific gene were given an additional point. These scores were summed for each gene, and genes with the same score were further prioritized based on the predicted number of targeting TSEs.

Loci are sorted first by overall score, secondarily by the number of STEs which are predicted to target the TSS based on GREAT.

Statistical significance for enrichment analyses was assigned using random permutation analysis (*n* = 10,000) with Benjamini-Hochberg correction for all tests.

Figures were generated using GOplot v1.0.2, ggplot2 v3.3.3, and ggsignif v0.6.1. ShinyCell (v.2.0.0) (80) package was used for development of the web portal. Detailed scripts of all analyses can be found at our github (https://github.com/cotneylab/scRNA_EnamelKnot).

This study makes use of data generated by the DECIPHER community. A full list of centres who contributed to the generation of the data is available from https://deciphergenomics.org/about/stats and *via* email from contact@deciphergenomics.org. Funding for the DECIPHER project was provided by Wellcome [grant number WT223718/Z/21/Z].

## Results

### Craniofacial enhancers contribute to dental development and phenotypes

**FIGURE 1:**
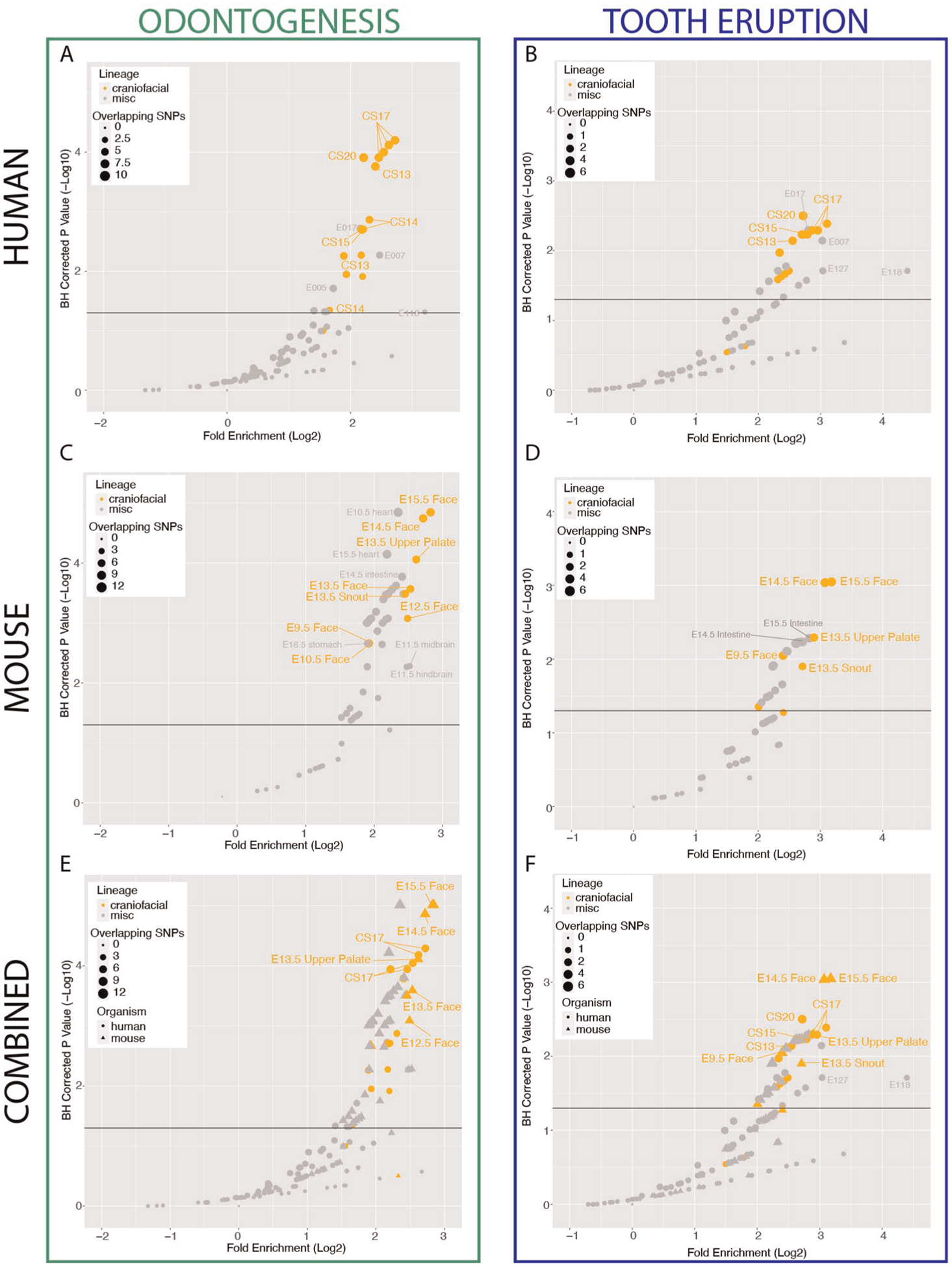
Conserved craniofacial enhancers contribute to dental development and phenotype risk. (**A**) Scatterplot of GREGOR (genomic regulatory elements and GWAS overlap algoRithm) analysis of enrichment of “Odontogenesis” (GO_0042476)-associated variants from GWAS Catalog in active enhancers annotated by 18 state chromatin segmentations genome-wide for samples from (40) (orange circles) and Roadmap Epigenome (grey circles). (**B**) Scatterplot of GREGOR (genomic regulatory elements and GWAS overlap algoRithm) analysis of enrichment of “Tooth Eruption” (GO_0044691)-associated variants from GWAS Catalog in active enhancers annotated by 18 state chromatin segmentations genome-wide for samples from (40) (orange circles) and Roadmap Epigenome (grey circles). (**C**) Analysis as in (**A**), with 18-state segmentations from Mouse ENCODE craniofacial (orange circles) and other mouse tissue samples (grey circles) (40, 51, 56). (**D**) Analysis as in (**B**), with 18-state segmentations from Mouse ENCODE craniofacial (orange circles) and other mouse tissue samples (grey circles) (40, 51, 56). (**E**) Co-projected plots of (**A**) (circles) and (**C**) (triangles), (**F**) Co-projected plots of (**B**) (circes), and (**D**) (triangles).

It has been well described that the regulatory regions which are strongly active in early craniofacial development play an important role in orofacial clefting and normal human facial variation (39–41, 81). Given the demonstrated genetic component of nonsyndromic odontogenic phenotypes such as delayed tooth eruption and hypodontia, we hypothesized that craniofacial regulatory regions may also play a role in these relatively common phenotypes. To test this, we compiled a list of “odontogenesis”-associated common variants from the GWAS catalog (*n* = 32; GO_0042476, “Number of Teeth”, “Time to First Tooth Eruption”; Supplementary Table S1) (82–88). We interrogated the enrichment of these dental phenotype-associated variants (DVs) within previously generated strong enhancers of the developing human face (CS13-CS20; 5–7 weeks post conception) (40) compared to strong enhancers of hundreds of other tissues (51), using a method which controls for population genetic structure and includes common variants which are within linkage disequilibrium with the reported lead variant (Methods) (60). We observed an enrichment of variants associated with odontogenic phenotypes in active enhancers of human embryonic craniofacial tissues, compared to hundreds of other tissue types from the Roadmap Epigenome Project (Figure 1A). We additionally investigated the genome-wide enrichment of variants associated with a subset of these SNPs specifically associated with Tooth Eruption (*n* = 14, GWAS Catalog GO_0044691, “Permanent Tooth Development”, “Primary Tooth Development”; Supplementary Table S2), which revealed a similar trend (Figure 1B). Notably, enrichment is not observed in craniofacial enhancers for variants associated with diseases not known to have a craniofacial component, such as Crohn’s disease (EFO_0000384) (Supplementary Figure S1). Taken together, these results suggest a role for the enhancers that are strongly active in the developing human craniofacial apparatus in human dental developmental traits.

Since key aspects of human dental development occur *in utero* it is challenging to obtain tissue and extremely difficult to perform experiments with frozen primary tissue samples that are available (40, 43–45). Thus the mouse has become a widely used model for key aspects of tooth development. While many features of tooth development are conserved between mouse and human, the number and shape of the teeth between these species are considerably different. Therefore it was unclear if the genetic enrichments we observed in bulk human craniofacial would translate to mouse regulatory regions. To address this, we leveraged hundreds of publicly available ChIP-Seq data sets collected by the Mouse ENCODE project from multiple mouse tissues across multiple stages of development, including the developing craniofacial prominence (56). We applied the same imputation and chromatin state segmentation approach used to identify human enhancers to the mouse data to generate 154 individual chromatin state annotations. We found that this approach identified 49,990 unique, reproducible putative mouse craniofacial enhancers spanning E10.5–E15.5. These enhancers were systematically enriched near genes known to be involved in craniofacial development and disease, mirroring results we reported for human craniofacial tissue (Supplementary Figure S2) (40).

We next identified strong enhancers from all mouse tissues which were conserved at the sequence level in humans, allowing us to systematically determine enrichment of the same odontogenic variants across mouse tissues (Methods). Similar to human craniofacial enhancers, we observed a specific enrichment of odontogenesis (Figure 1C) and tooth eruption (Figure 1D) associated variants in conserved mouse craniofacial enhancers above all other mouse tissues. Notably, when we directly compared the enrichment of these variants in the human craniofacial enhancers to the conserved mouse craniofacial enhancers, we observed the highest enrichment in conserved mouse craniofacial regions from stages where the developing teeth are known to be present and an appreciable percentage of the craniofacial apparatus, in particular E13.5–E15.5 (Figures 1E,F). It is important to note that the predicted equivalent human stages of craniofacial development [after CS23 or 8 weeks gestation (42, 46)] which contain comparable odontogenic tissue have not been assayed. The combination of these findings in human and mouse support our hypothesis that some common, non-syndromic human odontogenic phenotypes may be influenced by variation in enhancers that are active during craniofacial development. Specifically, the duration of tooth eruption and number of teeth in humans may be directly impacted by distal gene regulatory sequences active in the developing craniofacial apparatus. These findings also indicate that these regulatory regions may affect conserved processes in orofacial development.

### Developing tooth enhancers and disease burden

**FIGURE 2:**
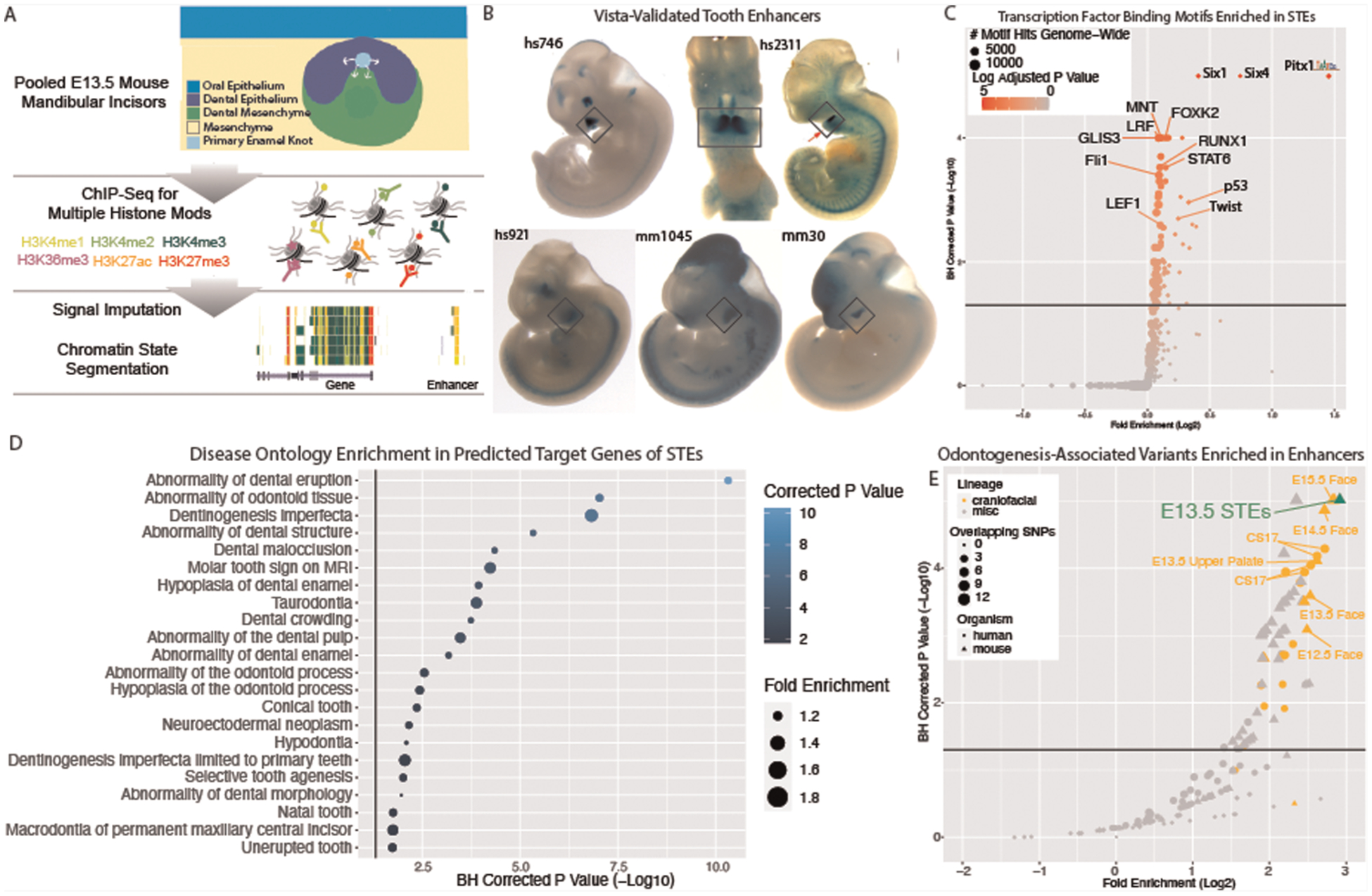
Enhancers of the developing tooth Are enriched for dental phenotype variants and target tooth development genes. (**A**) Schematic of ChIP-seq experiment and chromatin state annotation pipeline of E13.5 mouse mandibular incisors. (**B**) Selected VISTA-validated (58, 89) craniofacial enhancers which overlap TSEs. (**C**) Hypergeometric Optimization of Motif Enrichment (HOMER) (90) analysis of known transcription factor binding motifs enriched within tooth specific enhancers. Position weight matrices are demonstrated for each motif, obtained from JASPAR (133). (**D**) Dental disease ontology categories enriched in putative target genes of STEs, as assigned by Genomic Regions Enrichment of Annotations Tool (GREAT) (58). (**E**) GREGOR (60) analysis of enrichment of “Odontogenesis” (GO_0042476)-associated variants from GWAS Catalog in active enhancers annotated by 18 state chromatin segmentations genome-wide for samples from (40) (orange circles), Roadmap Epigenome (grey circles), and our own 18 state segmentations from ENCODE craniofacial ChIP-seq samples (orange triangles), E13.5 incisor (green) and other samples (grey triangles). Dotted line indicates log adjusted *p*-value threshold (1.3). Tooth specific enhancers (TSEs) do not pass significance threshold and cannot be evaluated for enrichment.

While these findings identified a role for regulatory regions in odontogenesis, they were based on bulk mouse and human craniofacial tissues composed of the developing tooth and also many other cell types. We hypothesized that this enrichment of odontogenic variants is contributed by enhancers active in the tooth, rather than enhancers active in the surrounding tissues, but there was no existing data to test this assumption. However, as discussed above, the scarcity and condition of primary human embryonic samples containing the tooth makes this very difficult. Therefore, we set out to experimentally identify enhancers active in the mammalian tooth in order to determine the role of enhancers contributed by the tooth rather than its surrounding tissue. The highest enrichment of odontogenic variants was observed in enhancers from bulk craniofacial samples spanning E13.5–E15.5, where the mandibular and maxillary teeth proceed through the cap stage. We looked to isolate cap stage teeth from an easily identifiable and accessible location, namely the mandibular incisors, at a time where the adjacent mesenchyme was not too mineralized to prevent manual dissection. Therefore, to identify enhancers active in the cap stage tooth, we isolated and pooled E13.5 mouse mandibular incisors and performed chromatin immunoprecipitation and sequencing (ChIP-seq) on a battery of post-translational histone modifications associated with a variety of functional chromatin states across the genome. This data was then analyzed using a standardized data analysis pipeline incorporating ChIP signal imputation and chromatin state segmentation, making it directly comparable to the previous human and mouse functional chromatin annotations (Figure 2A; Methods) (40, 51, 52). We identified 134,875 putative enhancers, of which 25,358 were predicted to be strong enhancers in the tooth (STEs). When we compared these strong enhancers to hundreds of other mouse samples, we identified 6,236 enhancers which were unique to the developing tooth (tooth-specific enhancers, TSEs; Methods). To confirm we had identified bona fide enhancers, we interrogated the genome-wide overlap of STEs with validated VISTA enhancers (Figure 2B) (89). We observed a significant enrichment of VISTA validated craniofacial enhancers within our STEs (*p* < 2.2 × 10^−16^; odds ratio = 3.55, Fisher Exact Test). Coordinate files of all strong cap stage enhancers (Supplementary Table S3) and tooth-specific enhancers (Supplementary Table S4) are available along with an interactive table of VISTA-validated tooth specific enhancers on our website (https://cotney.research.uchc.edu/tooth/).

Enhancers which are active in a tissue often demonstrate enrichment of binding motifs for transcription factors that show dynamic expression during the development of that tissue (52). Therefore we looked to further validate these enhancers by interrogating STEs for enrichment of all available vertebral transcription factor binding motifs (Methods) (90). STEs displayed the greatest enrichment of binding motifs for canonical dental transcription factor families *Pitx, Runx, Twist, Six*, and *Tcf*, whose members are known to be highly expressed in the tooth (Supplementary Table S5, Figure 2C) (91–95). In addition to motif enrichment, previous publications have shown that enhancers active in a tissue are enriched near genes with important roles in function or specification of that tissue (52, 96, 97). Therefore, we looked to further validate tooth enhancers using predicted target genes of these regions. We assigned STEs to putative target genes and interrogated these genes for enrichment of gene and disease ontologies (Methods). We observed significant enrichment for tooth-relevant gene and disease ontologies, including known dental developmental diseases such as abnormality of tooth eruption, taurodontia, and abnormal tooth number (Figure 2D). The presence of many canonical dental developmental genes both as STE targets and enriched binding motifs in STEs serves to further validate our cap stage tooth enhancer annotations. TSEs demonstrate similar enrichments of transcription factor binding motifs and ontology categories of predicted target genes, indicating STEs and TSEs are found in similar genomic loci and bind similar transcription factor families.

With these positive results of the validity of STEs, we aimed to test our hypothesis that this enrichment of odontogenic variants is contributed by enhancers active in the tooth itself, rather than enhancers active in the surrounding tissues. To achieve this, we obtained the orthologous human coordinates of mouse tooth enhancers with at least 25% sequence conservation, resulting in 22,001 conserved STEs and 4,127 conserved TSEs. Despite their importance in the tissue, the relatively low number of conserved TSEs compared to all conserved STEs (~4,100 vs. ~22,000) prevents a reliable calculation of genome-wide variant enrichment in TSEs. We therefore interrogated STEs for enrichment of human odontogenic variants using the same approach implemented above. We observed the highest and most significant enrichment (2.84 log2 fold enrichment; padj = 5.66 × 10^−6^) of odontogenesis-associated variants within conserved STEs (Supplementary Table S6, Figure 2E). This was recapitulated for tooth eruption (3.28 log2 fold enrichment, padj = 4.4 × 10^−4^, Supplementary Table S7, Supplementary Figure S3). These findings indicated that enhancers strongly active in the developing tooth are likely important in odontogenesis and odontogenic phenotypes. These findings additionally suggest the developing mouse tooth is an appropriate model system for the role of enhancers in human odontogenic phenotypes. However, the large number of enhancers we identified genome-wide required prioritization.

In tandem, we sought to further confirm that this enrichment was due to enhancers from the tooth itself, rather than the surrounding craniofacial milieu. To computationally dissect this, we subtracted the TSEs from our previously identified reproducible craniofacial enhancer set, resulting in 46,708 general craniofacial enhancers (non tooth specific enhancers). We interrogated these general craniofacial enhancers for GO and DO enrichment of their predicted target genes, and noted a decrease in the enrichment of tooth-related ontology categories compared to the STEs and TSEs (Supplementary Table S8). For instance, the enrichment of “negative regulation of odontogenesis of dentin-containing tooth” decreased from 19.25 fold enrichment in TSEs and 3.97 fold enrichment in STEs to 2.61 fold enrichment in general craniofacial enhancers. Similarly, when we calculated the enrichment of human odontogenesis-associated variants within these general craniofacial enhancers, we observed a decrease of 3.2 fold enrichment in non-tooth enhancers compared to tooth enhancers. We observed a similar decrease in enrichment when we compared results for tooth eruption-associated variants (decrease of 5 fold enrichment). With these results, we conclude that risk of human odontogenic phenotypes is enriched within enhancers active in the developing tooth itself.

### Transcriptomic analyses reveal co-expressed networks of cell type specific odontogenic genes

As we have detailed above, enhancers operate by facilitating expression of target genes. We reasoned that genes predicted to be regulated by STEs might be previously unknown players in tooth development. Thus, we sought to prioritize putative odontogenic enhancers based on their predicted target genes. To achieve this, we analyzed transcriptomic data from the developing tooth in a threefold process: (1) Identification of genes differentially expressed in the cap stage tooth compared to the surrounding bulk craniofacial apparatus; (2) Identification of cell type specific genes of the cap stage tooth; and (3) Identification of genes that are co-expressed across tooth development with genes from 1 to 2.

**FIGURE 3:**
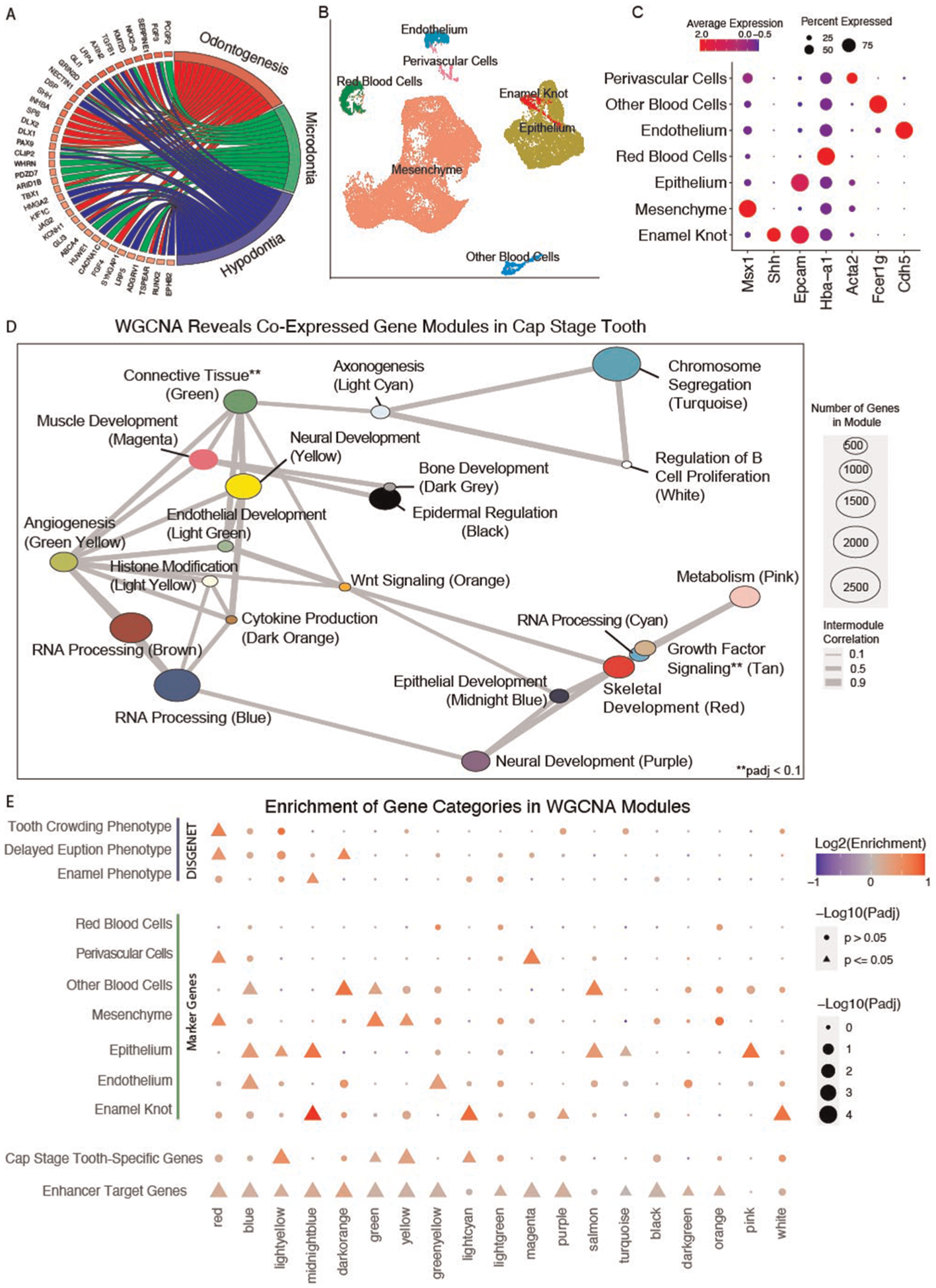
Weighted gene co-expression network analysis reveals dental development-specific coExpression modules. (**A**) Gene ontology map of genes differentially expressed in the cap stage tooth. (**B**) Annotated UMAP of cell types determined through Seurat analysis of 4 replicates of E14 mouse mandibular molars (*n* = 33,886 cells) from (62). (**C**) Dotplot of expression per annotated cluster from **B** for canonical dental cell type marker genes. Size is equivalent to percent expression, color is equivalent to expression. (**D**) Network plot of co-expressed modules of genes identified through WGCNA on embryonic mouse mandibular tooth samples from molars (E12.5–E17.5) and incisors (E12.5). A Pearson correlation of the module eigenvectors was calculated for the edges, and positive correlations of ≥0.5 were included. The location of each module is determined by multidimensional scaling (MDS) of the module eigengene vectors. Size of dots indicates the number of genes in each module, and colors are module names assigned by WGCNA. Modules are labeled according to module name and the most representative significantly enriched GO term of that module, as determined by clusterProfiler. (**E**) Permutation enrichment analysis (*n* = 10,000) of genes from each module within TSE target genes, and of human dental disease ontology categories, cap-stage differentially expressed genes, and marker genes identified from clusters in (**B**). Point size and shape indicates the significance of this enrichment, and color indicates the directionality of that enrichment (positive or negative).

To identify genes enriched in the cap stage mouse tooth, we leveraged published bulk RNA-seq data from the whole craniofacial apparatus (56) and isolated molars (62) at coordinating developmental timepoints (Methods). We observed 852 genes differentially expressed in the cap stage tooth compared to the bulk craniofacial apparatus (padj ≤ 0.05, log2FC ≥ 2, Supplementary Table S9). These tooth-biased genes were enriched in predicted STE target genes (1.28 log2 fold enrichment, *p* < 0.0001, permutation test), and predicted to be targeted by a median of 3 STEs per gene, and located a median of 93.3 kb away from their predicted enhancers. These differentially expressed genes were similarly enriched for predicted TSE target genes, with a median distance of 135.4 kb from TSE to the target gene. DEGs were also enriched for dentally relevant GO terms such as “Odontogenesis”, and human DO categories “Microdontia” and “Hypodontia” (Figure 3A). Together, these findings indicated that the identified tooth-biased genes are critical to odontogenesis, and tend to be located in proximity to, and are likely targeted by, enhancers preferentially active in the tooth.

We then looked to uncover genes enriched in the cell types of the cap stage tooth. We reprocessed published high quality scRNA-seq data from cap stage molars (Methods) (62). A graph-based clustering approach (98) revealed 33,886 cells separating into 16 transcriptionally distinct cell states (Supplementary Figure S4). Using pseudobulk methods (99) we identified 4,821 total marker genes, an average 301 per cluster. We leveraged significant GO and DO terms enriched in each cluster to assign biological identities per cluster (Figure 3B), and confirmed assignments with canonical marker genes (Figure 3C) (Methods). Notably, we identified an epithelial subcluster with attributes consistent with canonical features of the primary enamel knot (Supplementary Figure S5); we annotated these cells as the putative enamel knot (PEK, Figure 3B). Having annotated the cell types of the E14 mouse molar including the previously un-annotated PEK, we uncovered marker genes of each cell type. We identified 2,065 total unique marker genes, an average of 295 per cluster, including 317 PEK genes (Supplementary Table S10).

We then looked to validate the relative EK-specificity of PEK marker genes within the developing jaw, but the large number of genes made it necessary to rely on public *in situ* databases. We performed a literature survey and identified the GenePaint resource of *in situ* hybridization (ISH) images. This resource has systematically generated *in situ* hybridization data for over 17,000 genes across multiple stages of mouse development. The vast majority of these experiments were performed on E14.5 whole mount embryos, which is particularly relevant for our work here as the mandibular incisor and molar are at the approximate cap stage (74–77, 79) (Methods). Mining this database revealed 1,135 genes annotated as expressed in teeth at this stage in either NMRI or C57BL/6J mice. When we interrogated all maker genes in our analysis, we found 286 of 2,065 were annotated as expressed in the tooth and were significantly biased toward PEK genes over all other clusters (*n* = 77, 2.28 log2 fold enrichment, padj < 0.0001). However many of the PEK genes we identified had been assayed but not yet annotated for expression location by GenePaint. When we closely inspected the top 20% most highly enriched PEK genes (*n* = 63), which included 8 canonical EK genes and 55 genes not previously identified in the structure, we largely confirmed the single-cell RNA-Seq results. Specifically we found the majority of genes which had been assayed by GenePaint (49/58 assayed) showed strong and specific hybridization in the teeth closely mirroring the results for canonical EK markers *Shh, Wif1*, or *Wnt10b* (Supplementary Table S11, https://cotney.research.uchc.edu/tooth/). These findings strongly validated our *in silico* PEK assignments and supported them being *bona fide* EK genes.

While conventional differential gene expression is a powerful approach for identifying genes with strong changes in expression across biological conditions, it tests for each gene individually and independently of all other genes (65). Alternatively gene co-expression approaches explicitly try to identify expression relationships between multiple genes. These approaches have been shown to reveal biological and disease relevant modules of genes that have similar patterns of expression across developmental trajectories, have enrichment of cell type-specific gene, and contribute to tissue-relevant phenotypes (52, 100–102). Thus to gain a more comprehensive view of gene expression across tooth development, weighted gene coexpression network analysis (WGCNA) (68) was performed on publicly available bulk RNA-seq data from isolated molars and incisors from timepoints spanning initiation to mineralization of the tooth (Methods) (62, 63). This analysis identified 28 unique modules of coexpressed genes, spanning 22,016 expressed genes (Supplementary Table S12). These modules ranged in size from 63 to 5,330 genes with a median 361 genes/module. Many of these modules (19/28) were enriched for GO or DO terms relevant to biological processes in the developing tooth, and the interdependence of these processes is recapitulated in the inter-module correlation (Figure 3D).

To determine which WGCNA co-expression modules are likely specifically relevant for tooth development, we interrogated the WGCNA modules for enrichment of cell type specific gene signatures, and observed 14 modules with likely cell types of activity (Figure 3E). To further categorize these potentially tooth-relevant modules, we additionally interrogated them for enrichment of cap stage-specific genes, orthologs of known human odontogenic phenotypes, and predicted target genes of the putative STEs and TSEs (Methods). 19 of the modules demonstrated enrichment of at least one category (Figure 3E). As expected, we observed modules to be enriched for marker genes of cell types consistent with GO terms enriched in that module (Figures 3D,E). For instance, the midnight blue module was enriched for epithelial GO terms, marker genes of both the epithelium and the epithelial-derived PEK, and paralogs of genes associated with human enamel phenotypes. The red cluster demonstrates a similar finding, as it is enriched for skeletal GO terms, mesenchyme marker genes, and orthologs of genes associated with delayed tooth eruption in humans. Combined, these findings suggest that prioritized modules of co-expressed genes from bulk RNA-seq reflect cell type specific patterns of expression in the developing tooth. In particular, modules enriched for genes differentially expressed in the putative enamel knot are likely important to dental development.

### Data integration prioritizes dental enhancers at predicted odontogenic loci

These layers of transcriptomic data implicated several gene modules likely important to dental development. The genes within these modules as well as the regulatory regions controlling them are thus strong candidates for modulating odontogenesis in humans. We sought to leverage this data to inform our prioritization of candidate odontogenic enhancer loci for downstream analyses, integrating multiple data modalities (bulk and single cell transcriptomics, GWAS, ChIP-seq) to generate a prioritized list of loci. Therefore, genes were prioritized using the following general categories: (1) Differentially expressed in the cap stage tooth, (2) Predicted STE target, (3) scRNAseq marker gene, (4) Presence in a relevant WGCNA module, (5) Proximity of the human ortholog to a craniofacial-relevant variant from GWAS catalog (6).Number of predicted tooth specific enhancers, and (7).Proximity to VISTA-validated STEs (Methods). When we applied this filtering approach to 16,322 genes expressed in the developing tooth we prioritized 1,668 genes (Supplementary Table S13). These genes and their flanking noncoding landscapes are of particular interest for future investigations of odontogenic phenotypes. The top priority gene through this method was revealed to be *Runx2*, a canonical odontogenic factor with known roles in tooth morphogenesis and eruption in mammals (103–106). The top 20 genes (Table 1) also included known odontogenic genes *Twist1* and *Msx2* which cause phenotypes such as abnormal tooth morphology, abnormal mineralization, and tooth agenesis (107, 108).

**FIGURE 4:**
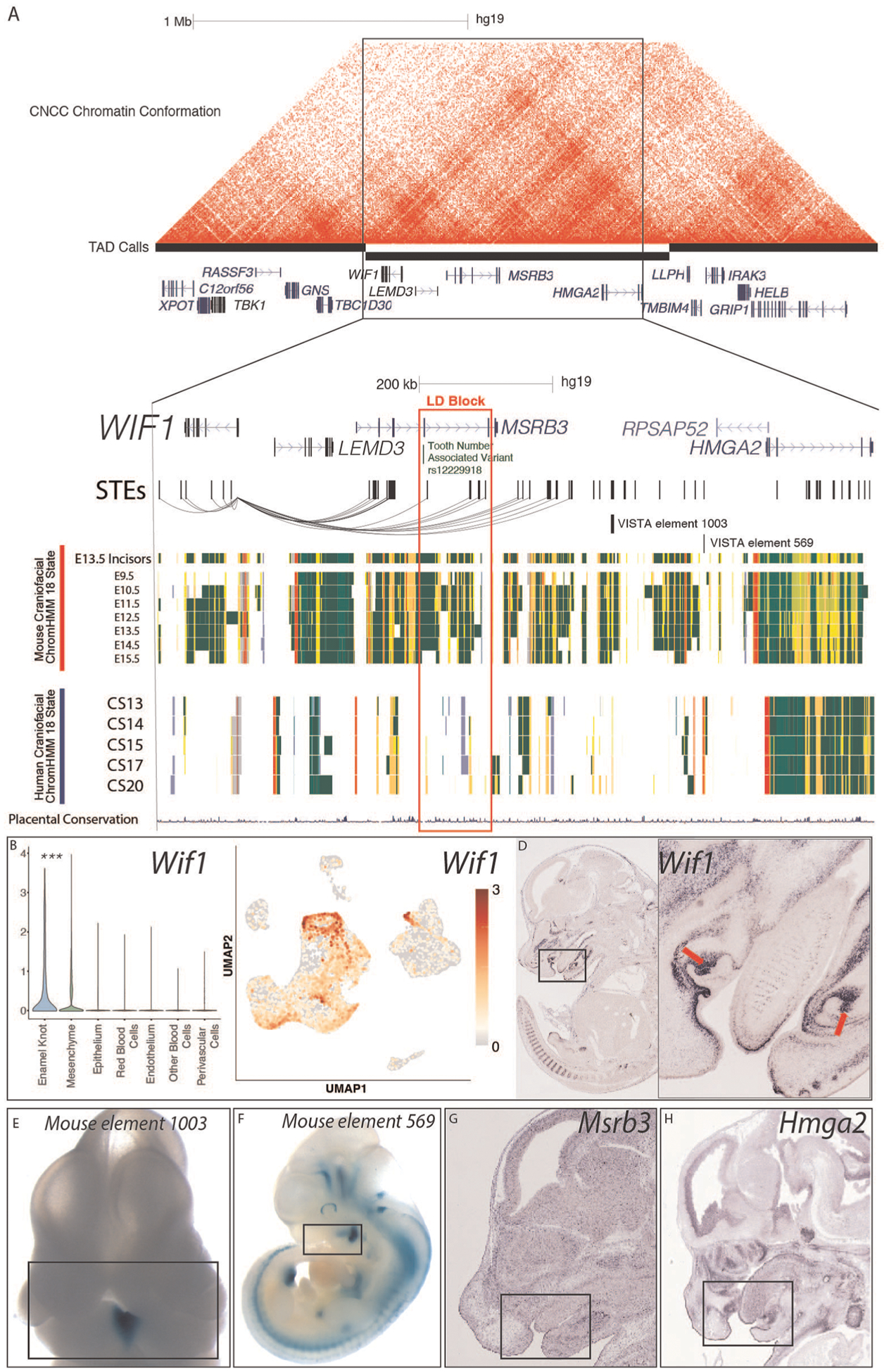
*WIF1* locus is a candidate for human odontogenic phenotypes. (**A**) Visualization of the locus surrounding rs12229918 (chr5:134509987, hg19) and nearby fine-mapped variants associated with decreased tooth number and delayed tooth eruption by (83). Chromatin conformation heatmap demonstrated using HiC from a human CNCC culture line, from (109). STEs are shown as hg19 coordinates. Mouse craniofacial 18 state segmentations were generated as described in Methods from data from ENCODE (56); these rows are from mm10 coordinates of the orthologous locus containing the orthologous genes. Human craniofacial 18 state segmentations were obtained from (40). (**B,C**) Single cell transcriptomic analysis demonstrated the PEK-specific expression of *Wif1* in the E14 mouse molar; *p* < 0.05, Wilcoxon test. (**D–G**) *In situ* hybridization images of *Wif1*, *Hmga2*, and *Msrb3* were obtained from GenePaint http://genepaint.org/viewer/setInfo/MH772, https://genepaint.org/viewer/setInfo/EN837, and https://genepaint.org/viewer/setInfo/EH300, respectively (75). Enhancer assay images were obtained from VISTA (89).

Also within the top 20 was *Wif1* (WNT inhibitory factor 1), a canonical enamel knot marker gene which was also annotated as a PEK marker in our single cell transcriptomic analyses. This locus demonstrates craniofacial enhancer activity in the mouse (Figure 4A), and includes many STEs which are predicted to target *Wif1*. When we inspected the *WIF1* genomic landscape in humans, we observed a variant associated with decreased tooth number and delayed tooth eruption (rs12229918) from human GWAS located in an intron of the upstream gene *MSRB3* (Figure 4A). Variant rs12229918 is within strong linkage disequilibrium of many other odontogenesis-associated variants which overlap human craniofacial enhancers, as well as conserved STEs from the developing tooth (83). Human chromatin conformation data from a human cranial neural crest cell culture line (109) (Figure 4A) demonstrates a topological associating domain (TAD) that includes the entire *WIF1* gene and multiple enhancers surrounding rs12229918. These findings suggested these conserved STEs directly target *WIF1* in a relevant developmental context in humans. This locus in both humans and mice contains *Wif1, Lemd3, Msrb3,* and *Hmga2;* of these, only *Wif1* demonstrates a tooth-specific or dental cell type-specific expression pattern in mice (Figures 4D,G; Supplementary Figure S6) (75). *Wif1* is known to play a role in normal mouse tooth morphology and be strongly expressed in the enamel knot, which we have confirmed in our scRNA-seq analysis (Figure 4) (110). While disruption of this gene has been associated with odontogenic phenotypes in mice, it has not been implicated in odontogenic phenotypes in humans. These findings suggest that variation at rs12229918 may alter a regulatory region interacting with *WIF1*, leading to elevated risk for nonsyndromic dental phenotypes.

**FIGURE 5:**
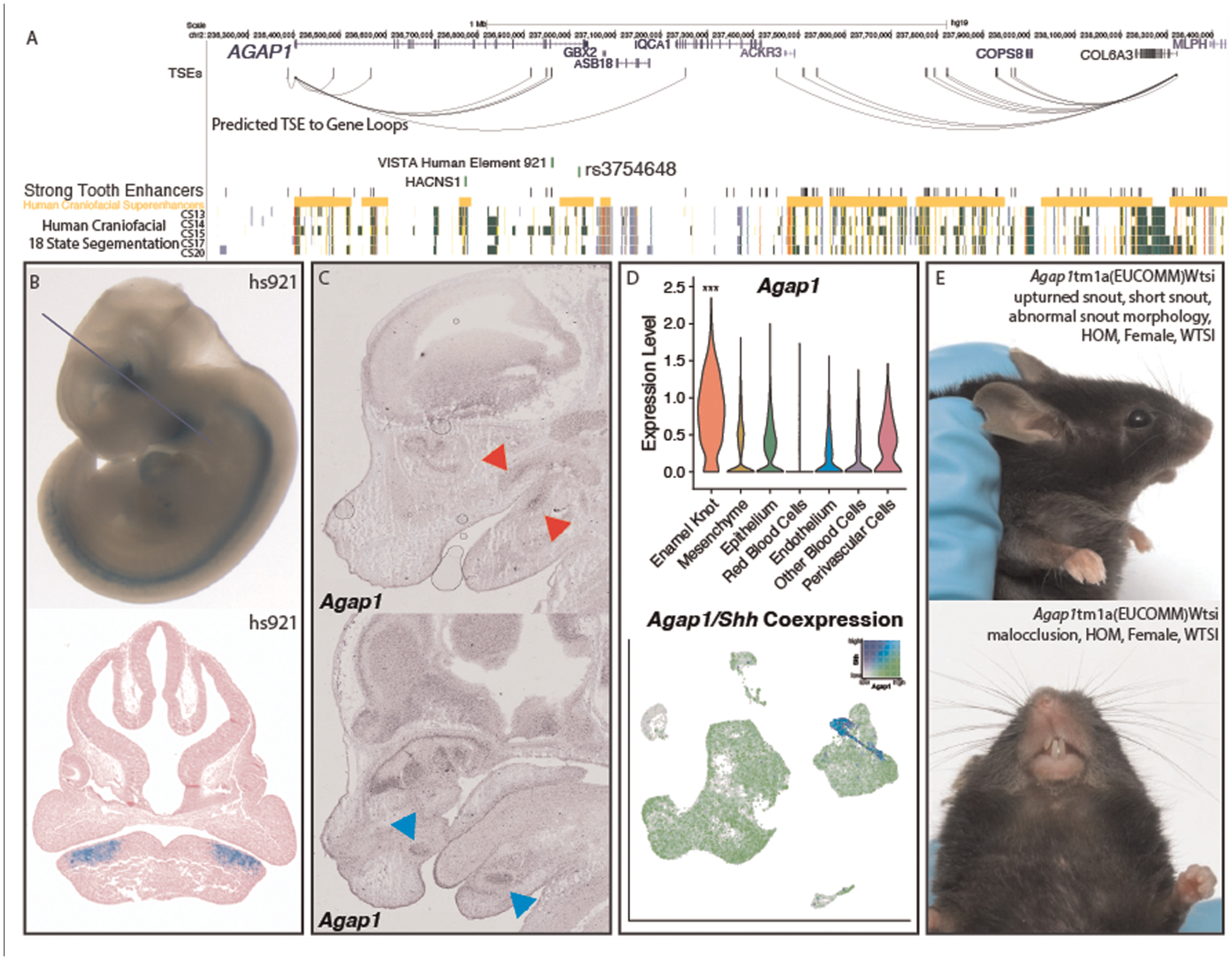
Validation of *Agap1* as a novel dental disease gene. (**A**) Visualization of the hg19 locus surrounding *AGAP1*. TSEs, strong tooth enhancers, and predicted TSE to gene loops were determined as described (Methods) using mm10 coordinates and lifted to hg19. (**B**) VISTA-validated enhancer hs921 demonstrates activity in the pre-mandibular pharyngeal arches, where it is limited to the approximate pre-dental regions. Images from VISTA (89). (**C**) *Agap1* expression is apparent in both the maxillary and mandibular molars (top, red arrows) and incisors (bottom, pink arrows). *in situ* hybridization images from GenePaint http://genepaint.org/viewer/setInfo/ES2973 (74–77). (**D**) Single cell transcriptomic analysis demonstrated the PEK-specific expression of *Agap1* in the E14 mouse molar. *p* < 0.05, Wilcoxon test. (**E**) *Agap1* loss of function mutations in mice result in malocclusion and abnormal facial feature phenotypes at 13 weeks postnatal age. Images from KOMP (111).

This finding at *Wif1* prompted us to look for other novel odontogenic genes in our prioritized list. We looked to profile high priority genes which had been assayed by the mouse knockout project (KOMP) without significant early lethality (111), and had not previously been described in the developing tooth. The highest priority novel odontogenic gene meeting these criteria was *Agap1* (Arf1 GTPase activating protein 1). *AGAP1* in humans contains the top scoring sequence of human acceleration on a conserved background in the genome, called HACNS1 (chr2:236773456–236774696) (112). This sequence is located in an intron of the gene, and actually lies within a human craniofacial superenhancer region (Figure 5A), suggesting its role in the craniofacial apparatus. Despite its proximity to known craniofacial regulatory regions, HACNS1 has been proposed to regulate *Gbx2* in the mouse limb (113). However, this gene is expressed at relatively low levels in human craniofacial tissues (97) and the mouse molar compared to *Agap1*. Additionally, *Agap1* is differentially expressed in the cap stage tooth compared to the bulk craniofacial apparatus. These findings suggest that *AGAP1* could play an important role in facial development and morphology and potentially be regulated in a human-specific fashion during tooth development. The 1Mb region surrounding *Agap1* contains many STEs and 7 TSEs which likely target *Agap1*, as it is the only tooth-specific gene in the region (Figure 5A). It is notable that while variants within the *AGAP1* protein-coding sequence have not been implicated in human disease, a variant intronic to *AGAP1* has been associated with hemifacial microsomia (rs3754648, *p* = 5 × 10^−13^), a disease which affects the patterning of the face and often coincides with dental phenotypes (114). This variant lies within a human craniofacial superenhancer region, and is within linkage disequilibrium of a conserved tooth-specific enhancer that was validated by VISTA (Figures 5A,B).

Despite the presence of a relevant variant within the gene, *AGAP1* has no reported dental phenotype in humans, and has not been associated with any specific human syndrome. Interestingly, the gnomAD entry for *AGAP1* demonstrates a LOEUF score (0.23) (115) in the bottom decile of all genes, indicating the gene is resistant to loss of function mutations in otherwise healthy individuals and therefore likely extremely important in humans. *Agap1* has also been demonstrated to be expressed in the upper and lower incisors and molars at E14.5 (74–77, 79), where it appears to be restricted to the previously described approximate enamel knot area (Figures 5C,D, Supplementary Figure S7). Supporting this finding is its high expression in the tooth compared to the bulk face, specifically in the PEK; *Agap1* appeared in our single cell analysis as a PEK marker gene, with 0.7 log2 fold enrichment in the PEK compared to all other cell types (padj = 1.51 × 10^−70^) (Figure 5D). Many genes specific to the EK result in dental phenotypes when knocked out specifically in the tooth (116, 117), and the specificity of this gene to the PEK within our data suggests there would likely be a similar phenotype. To determine if this is the case, we turned to the mouse knockout project (KOMP) to discern whether dental related phenotypes were apparent upon disruption of *Agap1*. Exploration of the database entry for the homozygous loss of function *Agap1* mice revealed clear and well described craniofacial phenotypes of snout malformation and malocclusion (improper meeting of maxillary and mandibular teeth) (Figure 5E, Supplementary Figure S8). Results from the KOMP also cited increased prevalence of “abnormal tooth morphology” (*p* = 9.12 × 10^5^).

Unfortunately these mice are not currently readily available from KOMP, precluding detailed analysis of the skulls and teeth. To begin to determine if *AGAP1* could be involved in human orofacial abnormalities we sought to investigate DECIPHER (118), a database of copy number variants in patients with developmental abnormalities. We found two patients with missense mutations in *AGAP1*, both were reported to have craniofacial and skeletal abnormalities no phenotypes related to teeth were mentioned. When we investigated patients with complete loss or gain of *AGAP1* (*n* = 140) we observed patients with a number of craniofacial abnormalities including low-set ears, micrognathia, and high palate. Interestingly, amongst these were 3 patients that were reported to have oligodontia. Oligdontia is reported to affect up to 0.2% of the general population and this is confirmed in the DECIPHER cohort overall with 79 patients reported to have oligodontia out of 31,843 total (0.25%). However 2.1% of patients with copy number variants spanning AGAP1 are reported to have oligodontia representing a 8.9 fold increase (*p* = 0.0052; two-tailed Fisher exact test) (119–125). Altogether, these findings suggest *AGAP1* is an unappreciated player in dental development in humans and mice, and that modulating its expression increases the likelihood of craniofacial and dental phenotypes.

## Discussion

Previous epigenomic findings in the bulk developing human craniofacial regions demonstrated a role for active regulatory regions in craniofacial morphology and disease. Here we have shown that regulatory regions active in the developing face likely also influence dental phenotypes and disease. Using publicly available ChIP-seq data (56) we generated functional chromatin annotations in hundreds of mouse tissues including craniofacial samples, which we used to demonstrate that conserved regulatory regions active in the early developing mouse face are similarly systematically enriched for variants associated with a human odontogenic phenotypes. We provide this resource of uniformly analyzed mouse genomic functional annotations spanning development of many tissues as an interactive UCSC genome browser session accessible from our website.

This role for enhancers in tooth phenotypes has not previously been demonstrated, and the role of enhancers in tooth development as a whole has been largely unexplored. Therefore, we performed ChIP-seq on isolated cap stage mouse incisors in order to understand the role of the enhancers from the tooth itself in these odontogenic phenotypes. We observed that this tissue demonstrates the highest enrichment of odontogenesis-associated variants, suggesting dysregulation of enhancers active in the tooth itself, rather than the surrounding tissues, contributes to these tooth-isolated phenotypes. These findings in regions conserved across species also suggest that the mouse tooth is a viable model for the role of enhancers in early dental development and certain odontogenic phenotypes. This investigation also served to identify over 25,000 strong enhancers in the tooth, including over 6,000 specifically active in the tooth and 22,000 enhancers conserved in humans. We have made the coordinates of these regions freely available in this manuscript and for download from our website, and included functional chromatin annotation in the UCSC session for enhancer visualization. To prioritize regions of these enhancers which may be contributing to these phenotypes, we prioritized their predicted target genes based on their likelihood to be relevant to tooth development. While integration of epigenomic and transcriptomic data has proven to be useful in identifying human disease candidate loci from developing tissue (52, 98), this method has not been applied to the developing mouse tooth, nor has it been extrapolated to indicate loci potentially associated with human odontogenesis.

In this manuscript we have also presented a re-analysis of publicly available scRNA-seq data from the E14 mandibular molar, from which we have identified a novel putative enamel knot gene signature. Using canonical EK marker genes, we annotated a previously undiscovered epithelial subcluster with features consistent with the EK (Supplementary Figure S4). We uncovered 317 PEK-specific genes and demonstrated the relative EK specificity of the majority of the 60 most specific PEK genes by comparing their expression within the approximate cap stage mandibular molar to *in situ* patterns of canonical EK markers *Shh, Wnt10a, and Cdkn1a* (Supplementary Table S8). By leveraging the GenePaint database of systematically generated E14.5 whole mount *in situ* images, many of which had several replicates, we were able to validate the relative specificity of these genes to the EK in the E14.5 mandible. Given the known role of the structure in patterning and morphogenesis of the tooth (126–128), this novel PEK signature suggests targets for future studies in dental regeneration and morphology, and is available on our website as an interactive table for further exploration.

Additionally, we have here demonstrated the ability to extrapolate multimodal data from the developing mouse tooth to human dental development and malformations. Previous investigations in human tooth development have been limited due to scarcity of the tissue, but they demonstrated the conserved expression of important dental factors such as RUNX2, WNTs, and BMPs (42–46). Many of these genes appear as cap stage tooth-specific genes, marker genes for dental cell types, and high priority putative odontogenic genomic loci (Supplementary Table S10). This speaks to both the robust nature of previous human investigations and our analyses, and indicates the validity of this prioritized list of loci. Our detailed findings at the *WIF1* loci also suggest the validity of the mouse tooth as a model of enhancers’ role in human dental development and odontogenic phenotypes. After integration of all our data modalities in this investigation, this gene appeared in the top twenty of our list of candidate odontogenic loci. We observed a variant highly associated with decreased tooth number and delayed tooth development which lies upstream of *WIF1*, a known odontogenic factor. This variant marks a region of conserved tooth enhancers, many of which are predicted to target *WIF1* (Figure 4). While *WIF1* has not been directly implicated in human odontogenesis, it belongs to the *WNT* pathway which is critical for both normal tooth patterning and root formation in mice (105, 129–131). The association of both patterning and eruption defects with tooth enhancers predicted to target this gene suggests that there are enhancers at this locus which are active in a stage-specific manner in the tooth. We predict that the *WIF1* locus contains enhancers which are differentially activated in the tooth during patterning and root formation. Disruptions of these distinct regions would result in the two distinct phenotypes of abnormal tooth number and eruption, respectively. Overall, *WIF1* serves as an example locus for conserved odontogenic enhancers likely modulating a conserved odontogenic gene whose dysregulation may result in malodontogenic phenotypes in humans.

Lastly, we have used our integrated data to profile a novel putative odontogenic gene, *AGAP1*. This gene has not been associated with any human dental developmental abnormalities, and has not previously been assayed in the mammalian tooth. Our prioritization process suggests that *AGAP1*, differentially expressed in the tooth and specifically expressed in the enamel knot, is a critical factor to mammalian odontogenesis. These findings were supported by the finding that mice deficient in *Agap1* demonstrate abnormal dental phenotypes (Figure 5, Supplementary Figures S5, S6). However, the lack of detailed descriptions of these phenotypes prevented any specific interpretations (i.e., enamel defects, cusp pattern differences) (111). Interestingly, the presence of the top scoring sequence of human acceleration on a conserved background in the genome (HACNS) within this gene suggests there may be a human-specific difference in the role for *AGAP1* in tooth development. These results together indicate *Agap1* as a novel odontogenic gene which is an attractive target for further investigations, particularly of species-specific mechanisms.

Further downstream experiments and analyses will be necessary to unravel the specific roles of prioritized candidate regions of enhancers in odontogenic phenotypes. Additionally, the tooth-specific enhancers we have identified could be attractive molecular tools for driving highly specific expression of reporter genes or Cre recombinase as we have shown for precise regions in the mouse brain (53). We provide a convenient portal to explore all of our results at https://cotney.research.uchc.edu/tooth/ with minimal computational effort for the convenience of the research community.

## Contribution to the field statement

Tooth developmental problems, such as abnormal tooth number and delayed tooth eruption, affect millions of people worldwide. These problems can require extensive and expensive dental treatment including braces, surgery, or implants. Additionally, they can increase risk of developing dental diseases and their complications. While some of these dental developmental problems appear as part of syndromes, such as orofacial clefting, many of these cases happen without affecting the rest of the body. These cases have not been linked to any genes, though they seem to be inherited traits. We show here that these dental problems are likely not due to mutations within genes themselves, but rather the regulatory regions which control the expression of genes during dental development. We identified 22,001 conserved regulatory regions genome-wide in the cap stage tooth, producing the first complete list of enhancers at this stage in this tissue. We show that regulatory regions of the tooth specifically are likely causing dental phenotypes, and that these regions are likely conserved across mammalian species. By combining this epigenomic data with gene expression data, we were able to prioritize these regions based on their likelihood of activity in the developing tooth, and therefore their likely influence on tooth development.

## Supplementary Material

Figure S8

Figure S7

Figure S6

Figure S5

Figure S4

Figure S3

Figure S2

Figure S1

Table S13

Table S12

Table S10

Table S9

Table S8

Table S11

Table S7

Table S6

Table S5

Table S4

Table S2

Table S3

Table S1

## Figures and Tables

**TABLE 1:** Top 20 prioritized putative odontogenic regions.

Rank	Gene	Score	WGCNA module	DEG	Marker gene	Predicted number of targeting TSEs
1	Runx2	11	Red	Yes	Mesenchyme	10
2	Cxcl14	10	Green	No	Mesenchyme	10
3	Six2	10	Yellow	Yes	Mesenchyme	6
4	Usp24	9	Salmon	Yes	No	15
5	Lmo4	9	Red	No	Perivascular Cells	13
6	Robo1	9	Purple	No	Enamel Knot	12
7	Tril	9	Lightgreen	No	Mesenchyme	10
8	Creb5	9	Blue	Yes	No	9
9	Bcl11a	9	Magenta	No	Epithelium	8
10	Gas1	9	Greenyellow	No	Mesenchyme	8
11	Sox11	9	Green	No	Epithelium	8
12	Twist1	9	Purple	No	Mesenchyme	8
13	Clic5	9	Green	No	No	7
14	Cxcl12	9	Green	No	Mesenchyme	7
15	Lrig3	9	Yellow	Yes	No	7
16	Msx2	9	Red	No	Enamel Knot	7
17	Rasl11b	9	Blue	No	Enamel Knot	7
18	Slc25a21	9	Green	Yes	No	7
19	Wif1	9	Red	No	Enamel Knot	6
20	Llph	9	Greenyellow	No	Epithelium	5

## Data Availability

The datasets presented in this study can be found in online repositories. The names of the repository/repositories and accession number(s) can be found below: https://www.ncbi.nlm.nih.gov/geo/query/acc.cgi?acc=GSE197645.
