# Supplementary figures and images for: Integration of multimodal data in the developing tooth reveals candidate regulatory loci driving human odontogenic phenotypes
Source: Front Dent Med. Author manuscript; Available in PMC 2023 Apr 6. (PMC10078798; doi:10.3389/fdmed.2022.1009264)

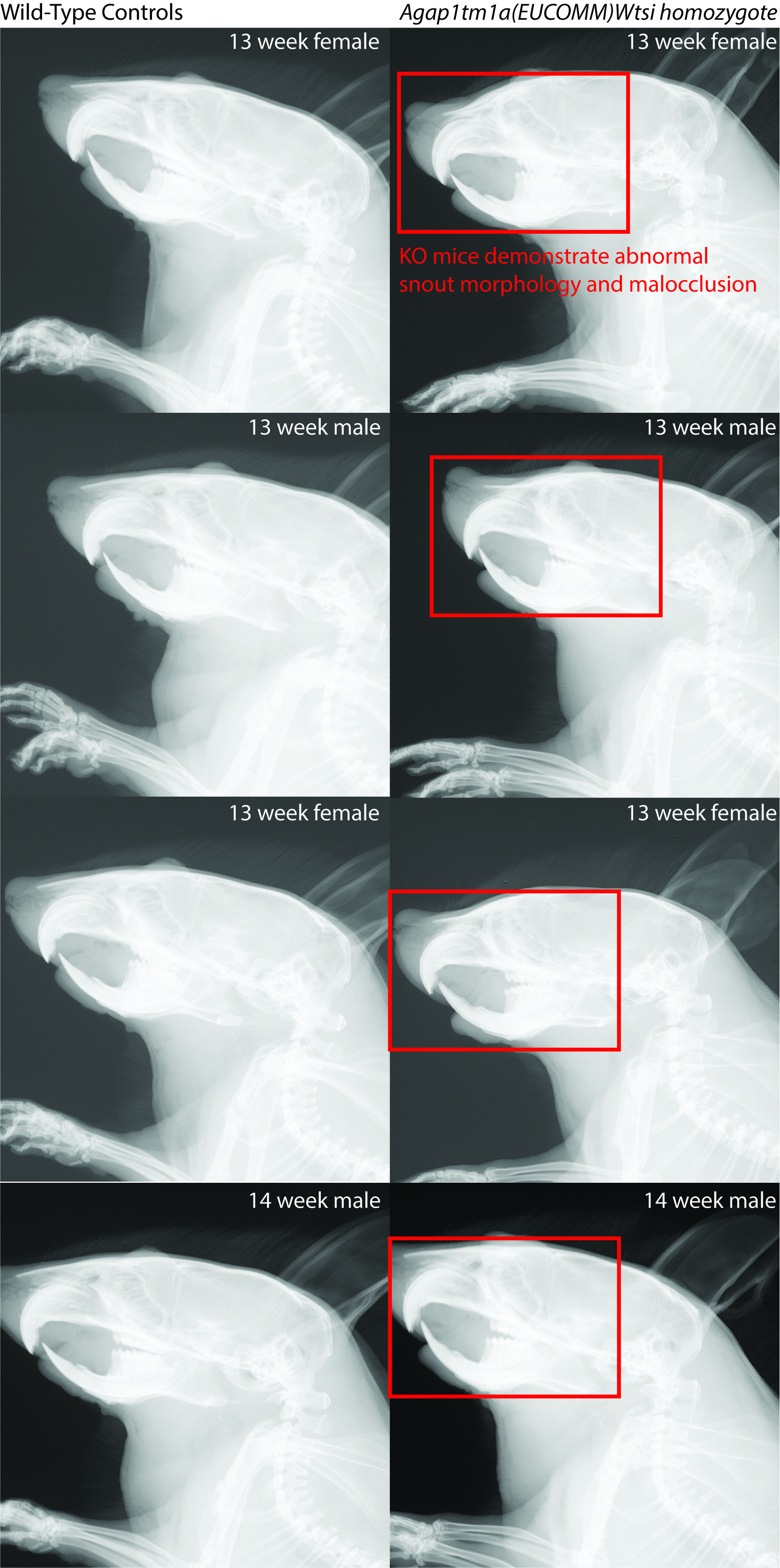

Supplement: Figure S8 [file NIHMS1880454-supplement-Figure_S8.jpeg]

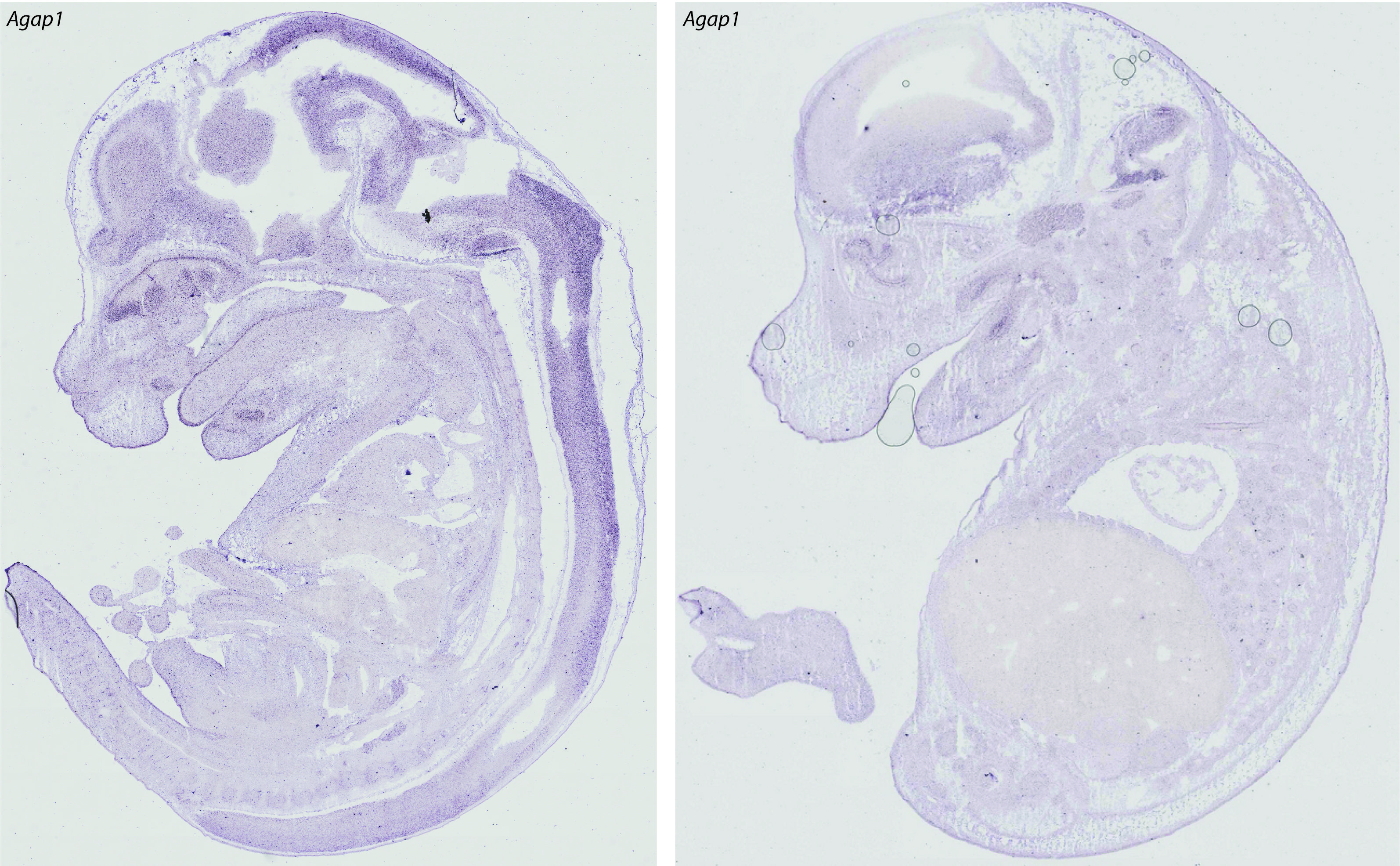

Supplement: Figure S7 [file NIHMS1880454-supplement-Figure_S7.jpeg]

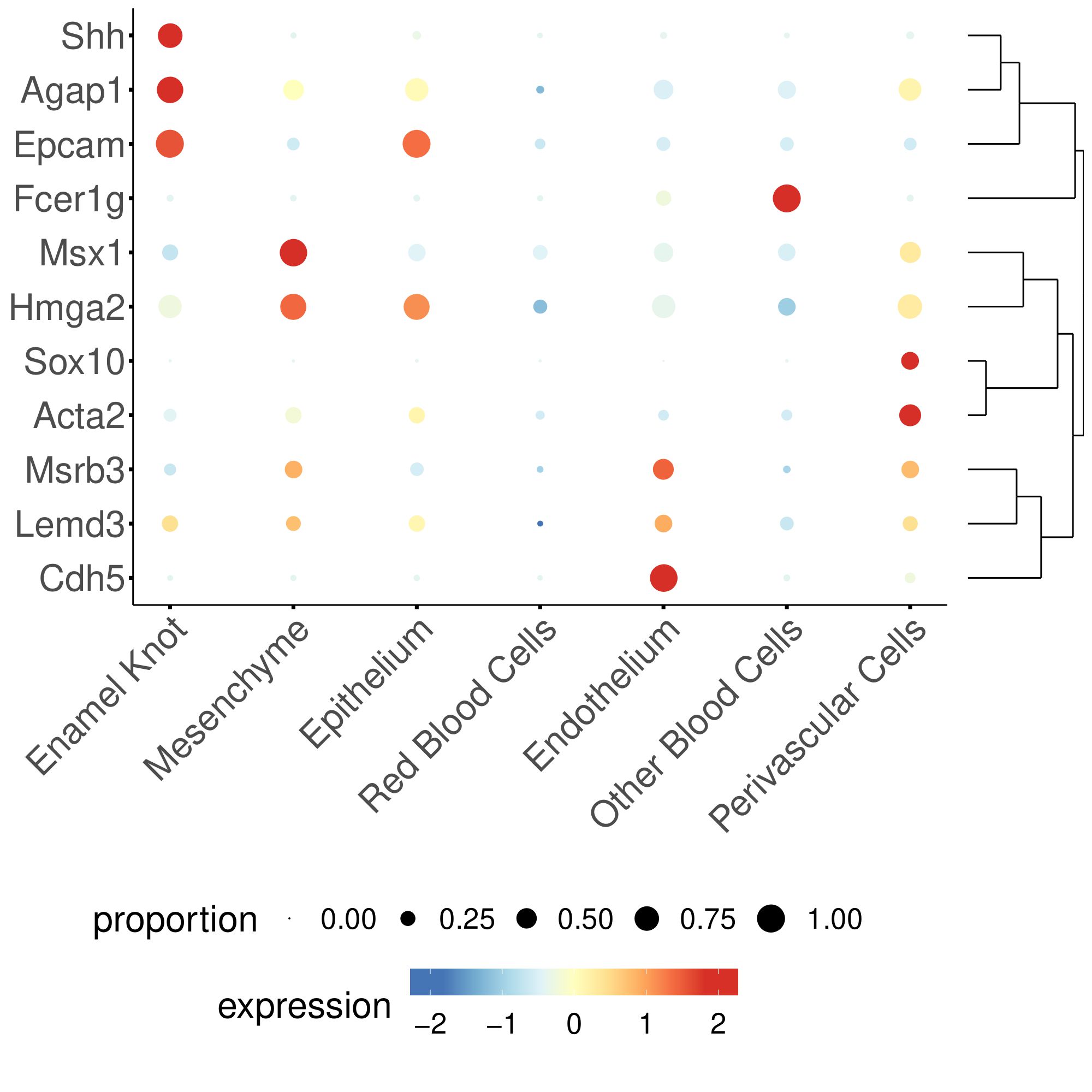

Supplement: Figure S6 [file NIHMS1880454-supplement-Figure_S6.jpeg]

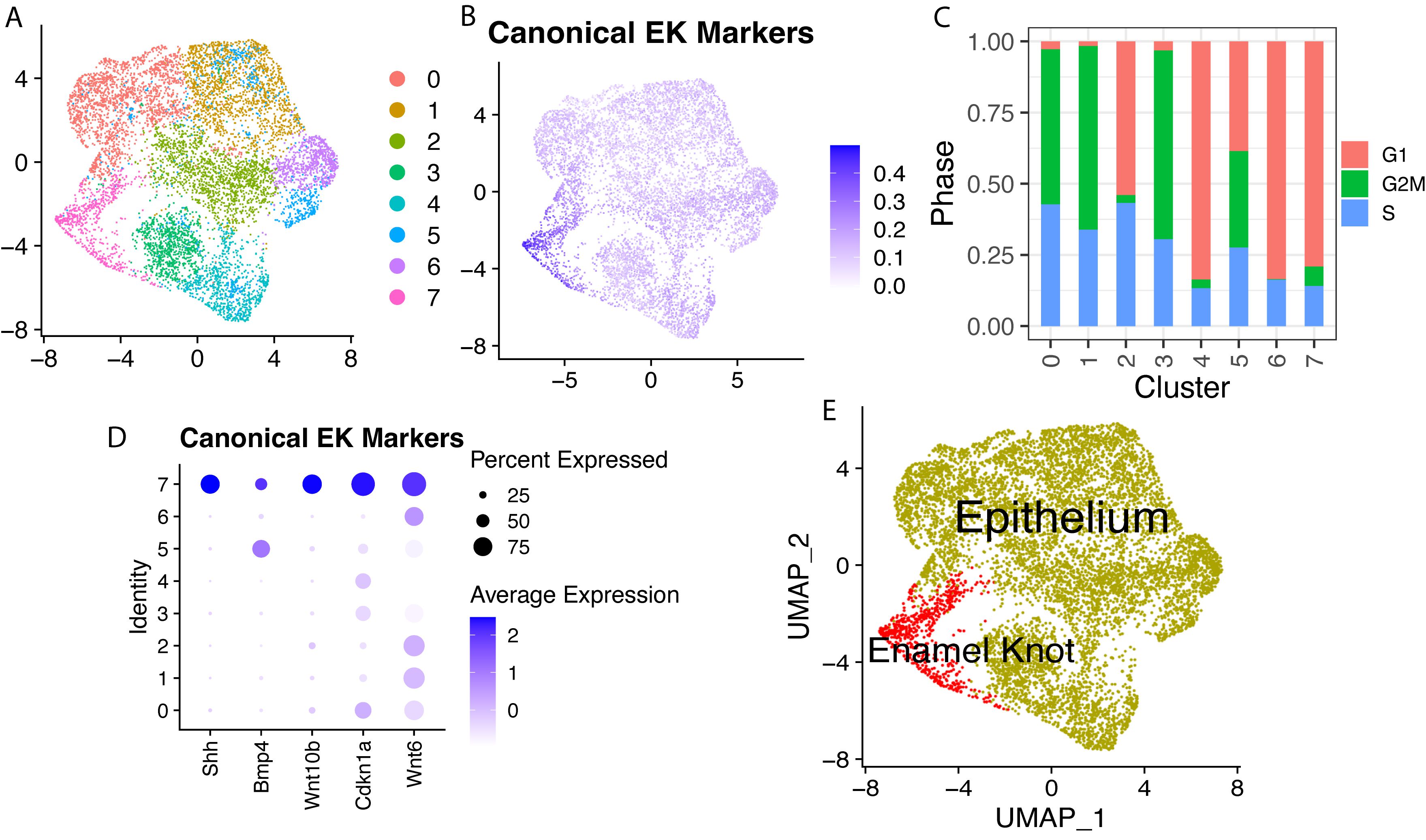

Supplement: Figure S5 [file NIHMS1880454-supplement-Figure_S5.jpeg]

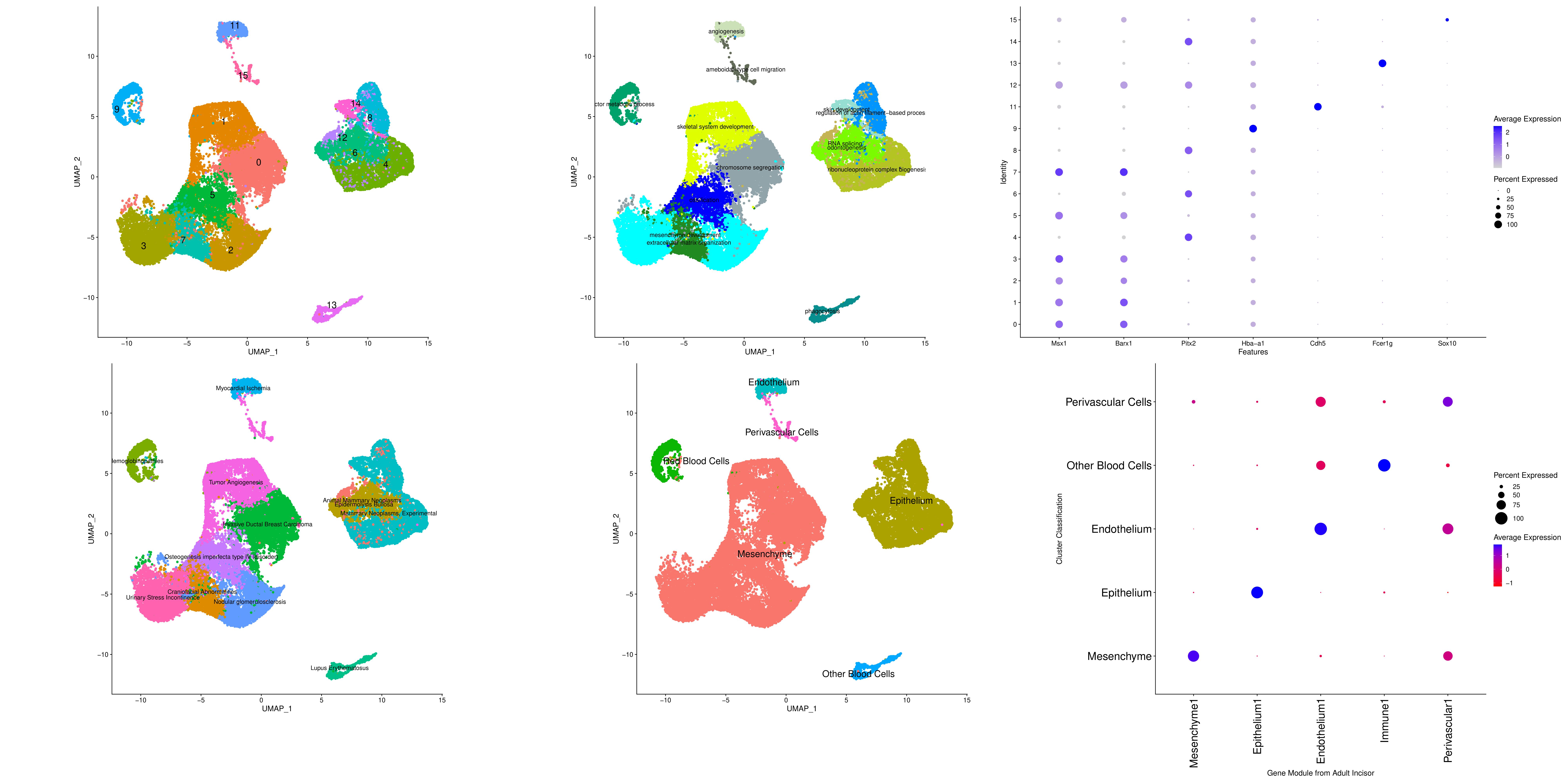

Supplement: Figure S4 [file NIHMS1880454-supplement-Figure_S4.jpeg]

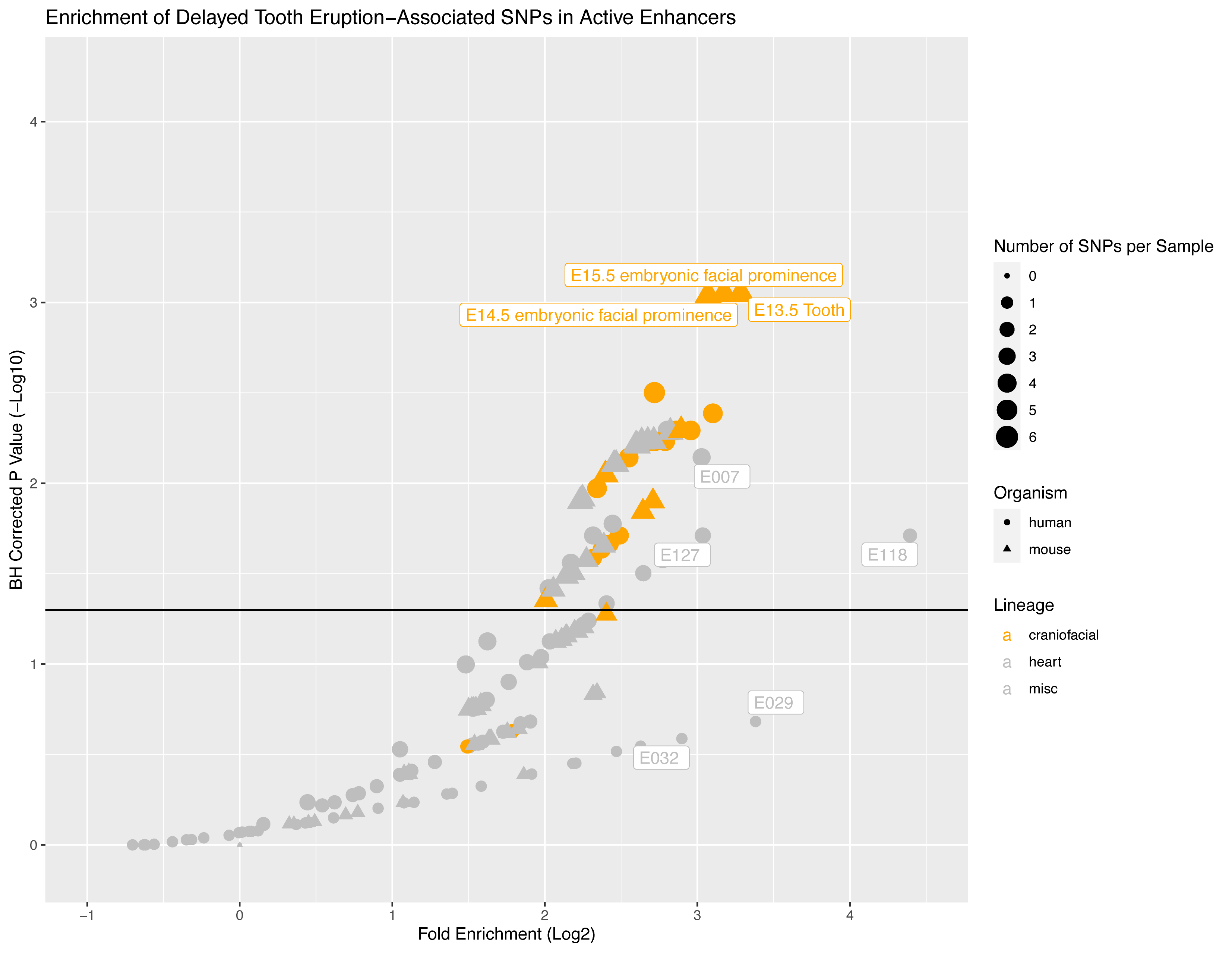

Supplement: Figure S3 [file NIHMS1880454-supplement-Figure_S3.tif]

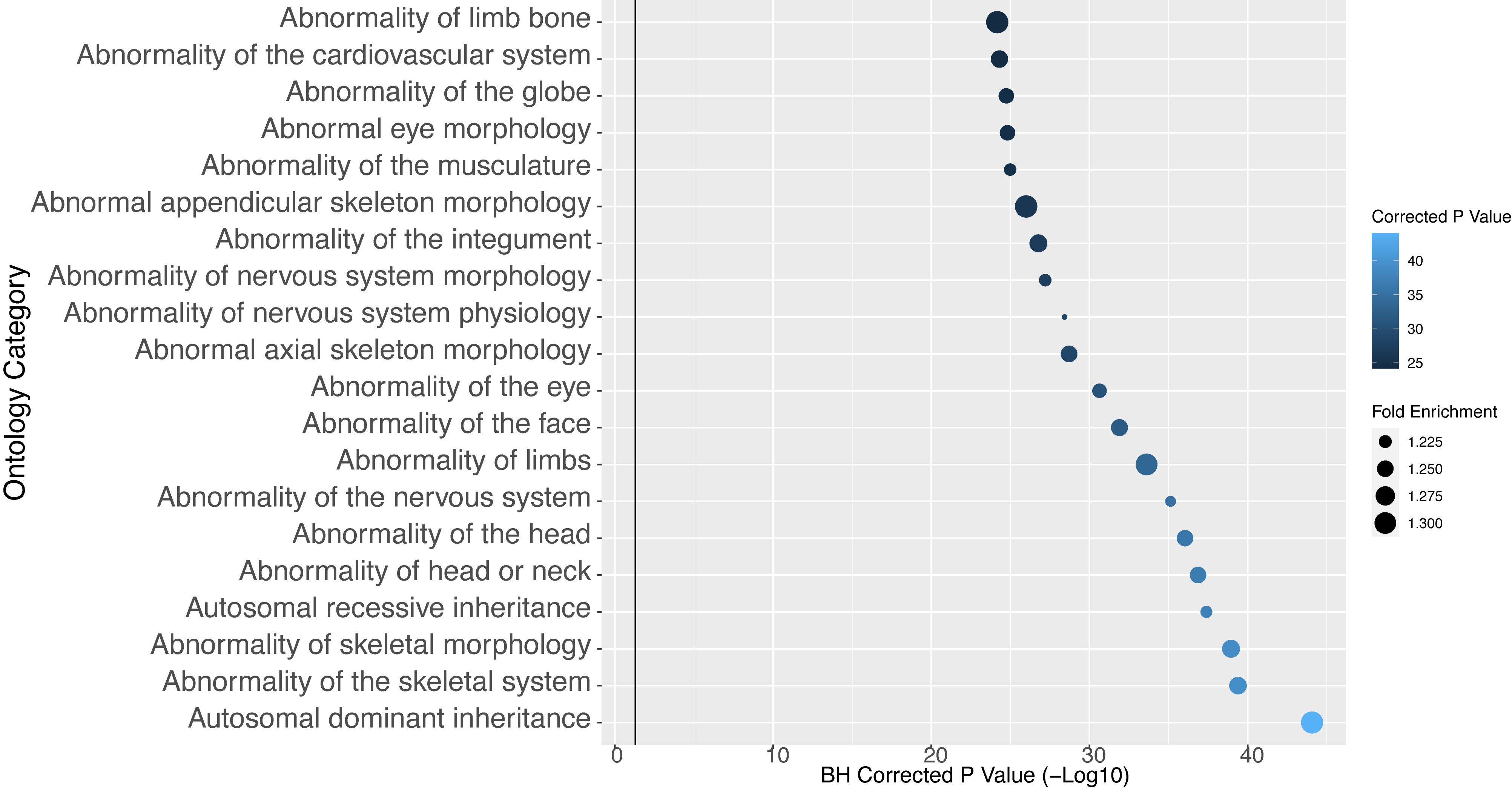

Supplement: Figure S2 [file NIHMS1880454-supplement-Figure_S2.tif]

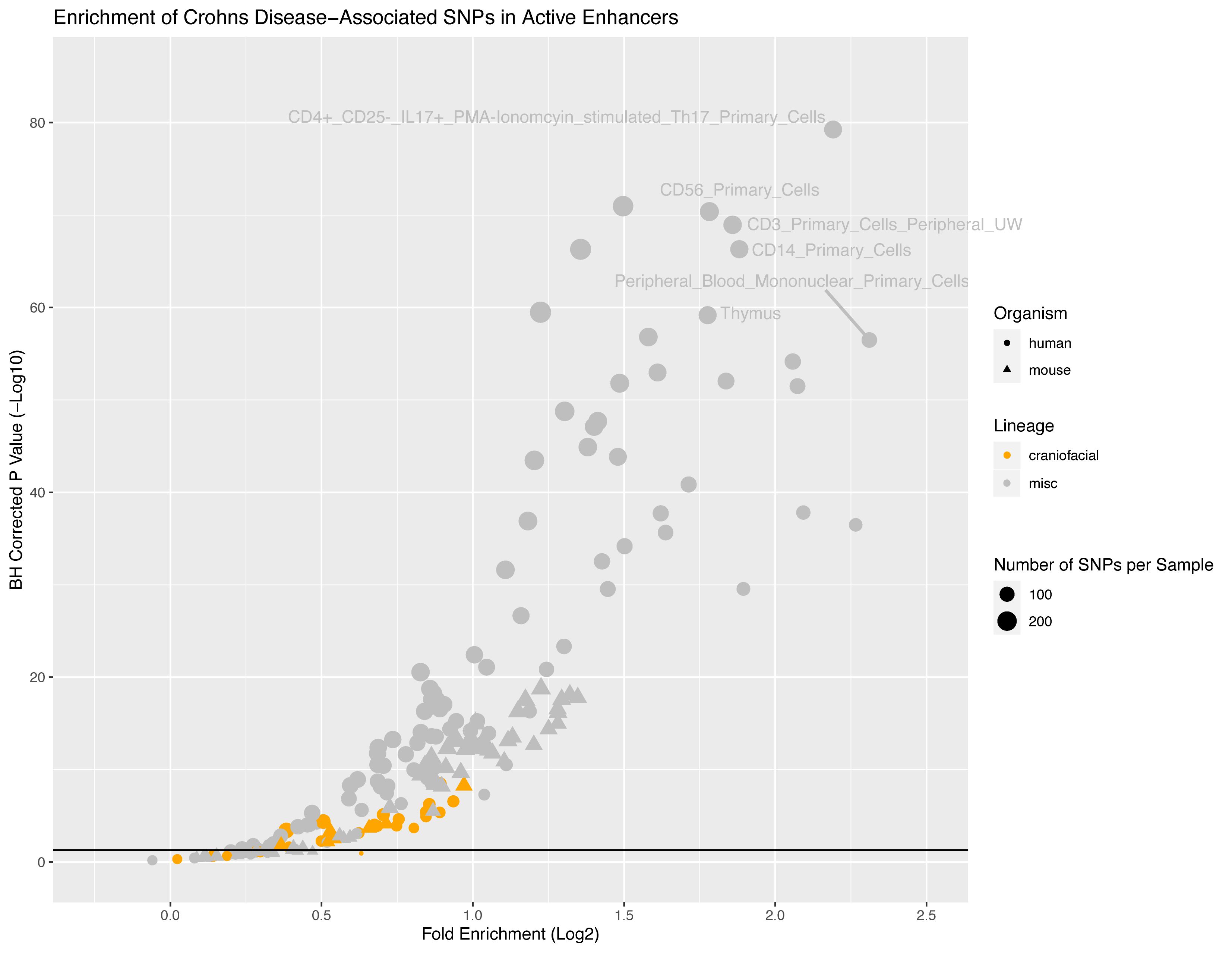

Supplement: Figure S1 [file NIHMS1880454-supplement-Figure_S1.jpeg]
